# Cryptomelane-Type KMn_8_O_16_ as Potential Cathode Material — for Aqueous Zinc Ion Battery

**DOI:** 10.3389/fchem.2018.00352

**Published:** 2018-08-17

**Authors:** Jiajie Cui, Xianwen Wu, Sinian Yang, Chuanchang Li, Fang Tang, Jian Chen, Ying Chen, Yanhong Xiang, Xianming Wu, Zeqiang He

**Affiliations:** ^1^School of Chemistry and Chemical Engineering, Jishou University, Jishou, China; ^2^The Collaborative Innovation Center of Manganese-Zinc-Vanadium Industrial Technology, Jishou University, Jishou, China; ^3^School of Energy and Power Engineering, Changsha University of Science and Technology, Changsha, China

**Keywords:** intercalated potassium compound, aqueous rechargeable battery, cathode material, energy storage and conversion, self-discharge

## Abstract

Aqueous battery has been gained much more interest for large-scale energy storage fields due to its excellent safety, high power density and low cost. Cryptomelane-type KMn_8_O_16_ confirmed by X-ray diffraction (XRD) was successfully synthesized by a modified hydrothermal method, followed by annealed at 400°C for 3 h. The morphology and microstructure of as-prepared KMn_8_O_16_ investigated by field-emission scanning electron microscopy (FE-SEM) with the energy spectrum analysis (EDS) and transmission electron microscopy (TEM) demonstrate that one-dimensional nano rods with the length of about 500 nm constitute the microspheres with the diameter about 0.5~2 μm. The cyclic voltammetry measurement displays that the abundant intercalation of zinc ions on the cathode takes place during the initial discharge process, indicating that cryptomelane-type KMn_8_O_16_ can be used as the potential cathode material for aqueous zinc ion batteries. The electrode shows a good cycling performance with a reversible capacity of up to 77.0 mAh/g even after 100 cycles and a small self-discharge phenomenon.

## Introduction

Although the lithium-ion batteries (LIBs) as one of the most promising energy storage devices have gained a great improvement in energy density and life span, and correspondingly dominated in the fields of portable mobile devices, electric vehicles (EVs) and hybrid electric vehicles (HEVs), the nonnegligible safety issues resulted from the flammable organic electrolytes seem to restrict their large-scale applications (Liu et al., 2014; Wang et al., 2015b; Su et al., 2018; Zhang et al., 2018). On the contrary, the aqueous rechargeable batteries can overcome these disadvantages mentioned above, which have attracted extensive attentions since the aqueous batteries of VO_2_/LiMn_2_O_4_ was firstly proposed by Dahn's group in 1994 (Li et al., 1994), especially the aqueous rechargeable batteries based on zinc anode, considering its multivalent characteristic, low cost, abundance and environmental benignity of zinc. However, up to now, only a few cathode materials have been developed as the intercalation hosts for metal ions. Rechargeable hybrid aqueous battery (ReHAB) system based on LiMn_2_O_4_ as the cathode and zinc as the anode was reported firstly by Chen's group, of which the capacity retention is up to 90.0% even after 1,000 charge/discharge cycles (Yan et al., 2012). After that, the similar systems such as Zn/LiMn_2_O_4_ (Lu et al., 2016; Zhu et al., 2016; Sun et al., 2017), Zn/LiMnPO_4_ (Minakshi et al., 2006), Zn/LiMn_0.8_Fe_0.2_PO_4_ (Zhao et al., 2016), Zn/LiCo_1/3_Mn_1/3_Ni_1/3_PO_4_ (Kandhasamy et al., 2012), Zn/LiFePO_4_ (Zhang et al., 2013), Zn/LiCo_1/3_Mn_1/3_Ni_1/3_O_2_ (Wang et al., 2015a) were reported. Nevertheless, the processing cost and the limited lithium resources result in a tremendous challenge for application. Thus, it is urgent for us to explore new non-lithium intercalation compounds as the cathode so as to match with zinc anode.

Among them, tunnel-type manganese oxides have been mostly investigated, including α-, β-, γ-, and δ-types MnO_2_ (Xu et al., 2012, 2014; Alfaruqi et al., 2015a,b,c; Pan et al., 2016; Han et al., 2017; Zhang et al., 2017). Although considerable initial discharge capacity up to 200 mAh/g at low *C*-rate can be delivered, they suffer from the poor rate performance and a rapid capacity fading owing to the repeated phase transitions and the dissolution of Mn^2+^ owing to Mn^3+^ disproportionation upon cycling. Moreover, the reaction mechanism of MnO_2_ remains controversial (Lee et al., 2015). A family of prussian blue analogs (abbreviated as PBAs) such as zinc hexacyanoferrate (ZnHCF) are also the attractive cathode materials based on zinc anode, which allow the rapid metal ion diffusion due to their cubic open-framework structures (Zhang et al., 2015a,b; Liu et al., 2016). However, these cathodes delivered the limited capacities (about 50 mAh/g) and suffered oxygen evolution under the high voltage.

One dimensional (1D) tunnel structured cryptomelane type manganese dioxides, Mn_8_O_16_ (α-MnO_2_) as the cathode materials have been received extensive concerns, as they can reversibly host various cations including Li^+^ and K^+^ and so on, of which K_x_Mn_8_O_16_ was previously reported in the fields of catalysis and lithium ion battery (Poyraz et al., 2017). Herein, this study aims to establish the aqueous hybrid battery based on cheap intercalated potassium compound KMn_8_O_16_ as the cathode material and zinc as the anode. We try to characterize the structure and its morphology and demonstrate its charge/discharge mechanism and excellent electrochemical performances.

## Experimental

Cryptomelane-type KMn_8_O_16_ was synthesized by a modified hydrothermal method (Poyraz et al., 2017). The typical preparation procedure is as follows, 6 mmol of MnSO_4_·H_2_O and 12 mmol of (NH_4_)_2_SO_4_ and 12 mmol K_2_SO_4_ were dissolved in 50 mL of 6 mmol (NH_4_)_2_S_2_O_8_ solution. After being magnetic stirred for 30 min, the solution was then transferred to a Teflon-lined stainless steel autoclave and was maintained at 130°C for 24 h. After it was naturally cooled to room temperature, the resulting product was washed with distilled water and anhydrous alcohol for several times, collected by centrifuge and followed by dried at 60°C overnight. Finally, it was annealed at 400°C for 3 h to obtain KMn_8_O_16_.

The phase and structure of as-prepared samples were identified by powder X-ray diffraction (XRD, D8 Discover, Bruker) employing Cu Kα (λ = 0.15406 nm) radiation from 10° to 65°. Morphology observation for KMn_8_O_16_ was conducted on a field-emission scanning microscopy (FE-SEM, Leo-1530, Zeiss) with the energy spectrum analysis (EDS) (accelerating voltage of 20 kV). The morphology and microstructure of the material was measured by transmission electron microscopy (TEM, Tecnai G12, 200 kV). The surface element analysis of the as-synthesized product was used by X-ray photoelectron spectroscopy (XPS, K-Alpha 1063), and then the spectra obtained was fitted with XPS peak software (version 4.1).

The working electrode was prepared by casting the slurries of 80 wt% KMn_8_O_16_, 10 wt% polyvinylidene fluoride (PVDF) and 10 wt % acetylene black on graphite foil. After being blended in N-methyl pyrrolidinone, the mixed slurry was spread uniformly on graphite foil and dried in a vacuum for 4 h at 60°C. Disks of 14 mm diameter were cut (the typical active material loading was about 2~3 mg/cm^2^) and soaked in hybrid electrolyte solution under vacuum for 15 min. AGM (Absorbed Glass Mat, NSG Corporation) was used as the separator. Then the electrochemical performances were evaluated using CR2032 coin-type battery based on zinc foil as the anode. The galvanostatic charge-discharge was performed by way of a battery tester (LAND in China) in the potential range of 0.8~1.9 V at room temperature, and the cyclic voltammetry curve was tested on an electrochemical workstation (CHI 660E).

## Results and discussion

**Figure 1:**
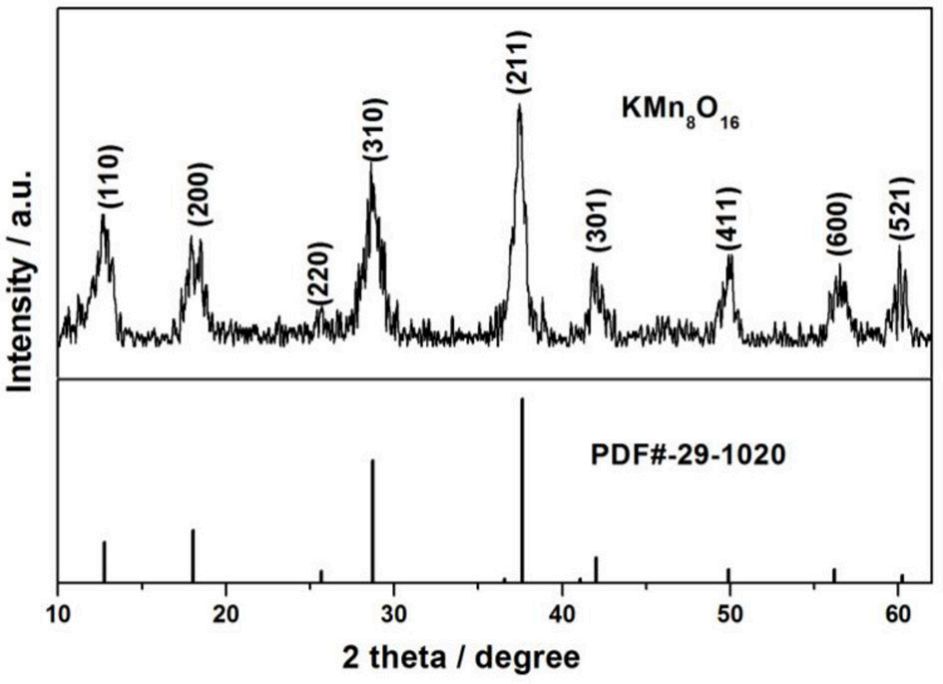
The XRD patterns of KMn_8_O_16_ sample.

**Figure 2:**
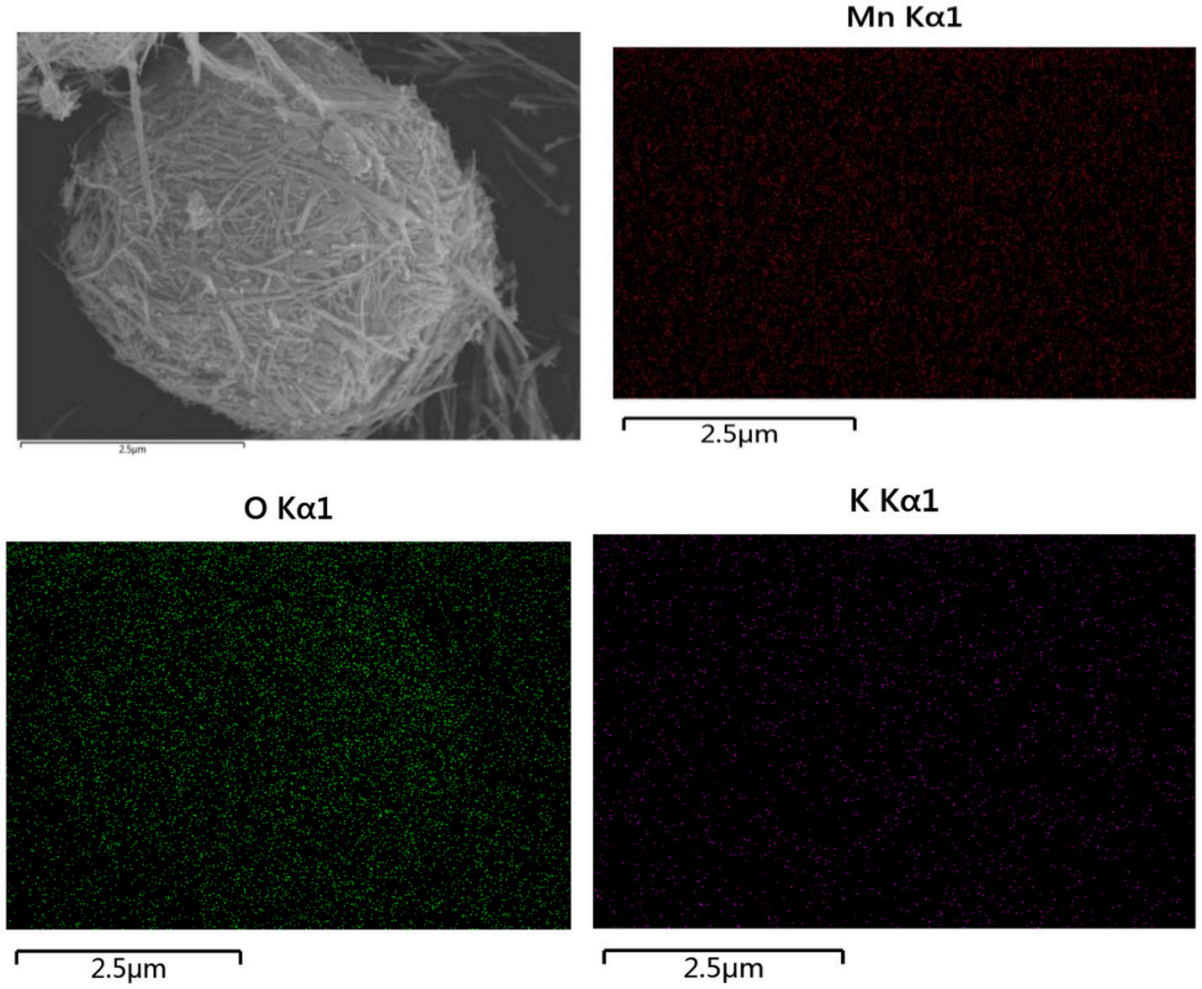
The elemental mapping images of KMn_8_O_16_ sample.

The crystalline structure of as-obtained products after heat treated at 400°C in air for 3 h was analyzed by XRD. It can be clearly observed from Figure 1 that all the diffraction peaks of the sample at 2θ = 12.745°, 18.059°, 25.651°, 28.737°, 37.62°, 42.029°, 49.896°, 56.184°, and 60.24°Can be readily indexed to (110), (200), (220), (310), (211), (301), (411), (600), and (521), which are in well agreement with the pure tetragonal cryptomelane structures of KMn_8_O_16_ (JCPDS card No. 29-1020; space group:*I*4/m(87), a = b = 9.815 Å, c = 2.847 Å, and α = β = γ = 90°), and the average crystal sizes of KMn_8_O_16_ determined by using the Scherrer formula (L = 0.89λ/βcosθ) at 2θ = 18.1° is 17 nm. Meanwhile, EDS elemental mapping in Figure 2 reflects the uniform distribution of K, Mn and O in the material, and there are no other impurity elements, indicating that K^+^ ions can be embedded into the interlayer space of MnO_2_ structure. These results demonstrate that KMn_8_O_16_ can be successfully prepared.

**Figure 3:**
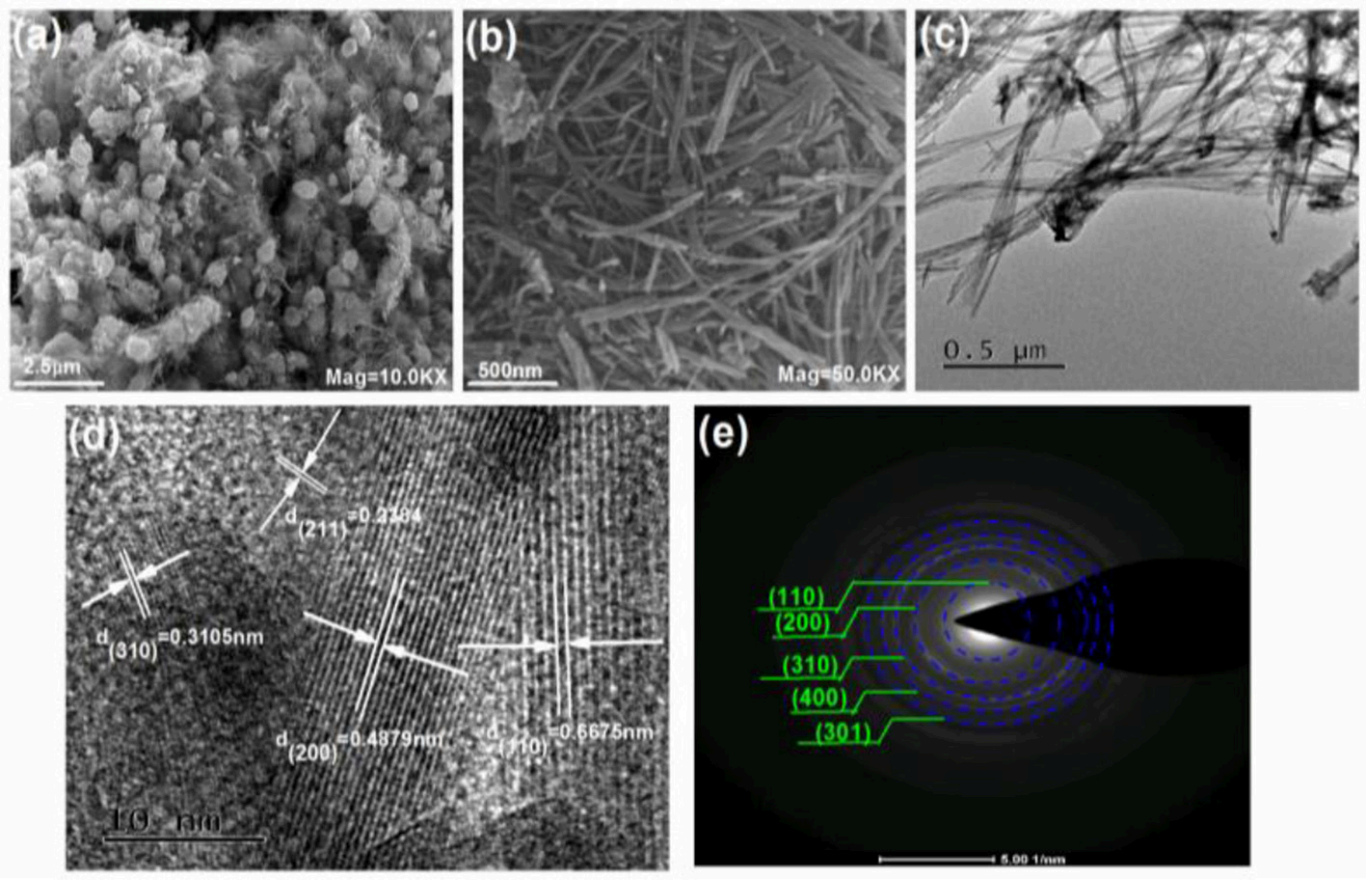
SEM images **(A,B)**, TEM image **(C)**, HRTEM image **(D)** and SAED **(E)** of KMn_8_O_16_ sample.

Furthermore, FE-SEM and TEM are used to demonstrate the morphology and microstructure of the as-prepared products in Figure 3. The results in Figure 3A show that the secondary particles of KMn_8_O_16_ exhibit the microspheres with the diameter about 0.5~2 μm. Meanwhile, it is carefully noted that many one-dimensional nano rods with the length of about 500 nm constitute the microspheres in Figure 3B. Further the details of the structural characteristic are indicated by TEM. The primary particle is made up of nano rods interconnected each other in Figure 3C, and the lattice fringes with the interplanar acing of 0.2384, 0.3105, 0.4879, and 0.6675 nm from the HRTEM images in Figure 3D are assigned to the (211), (310), (200), and (110) planes of KMn_8_O_16_, respectively. No other diffraction peaks of impurities have been detected. Meanwhile, the selected area electron diffraction (SAED) pattern in Figure 3E corresponds to the characteristic diffraction rings of (110), (200), (310), (400), and (301) planes of tetragonal KMn_8_O_16_, all of which agree well with the XRD results.

**Figure 4:**
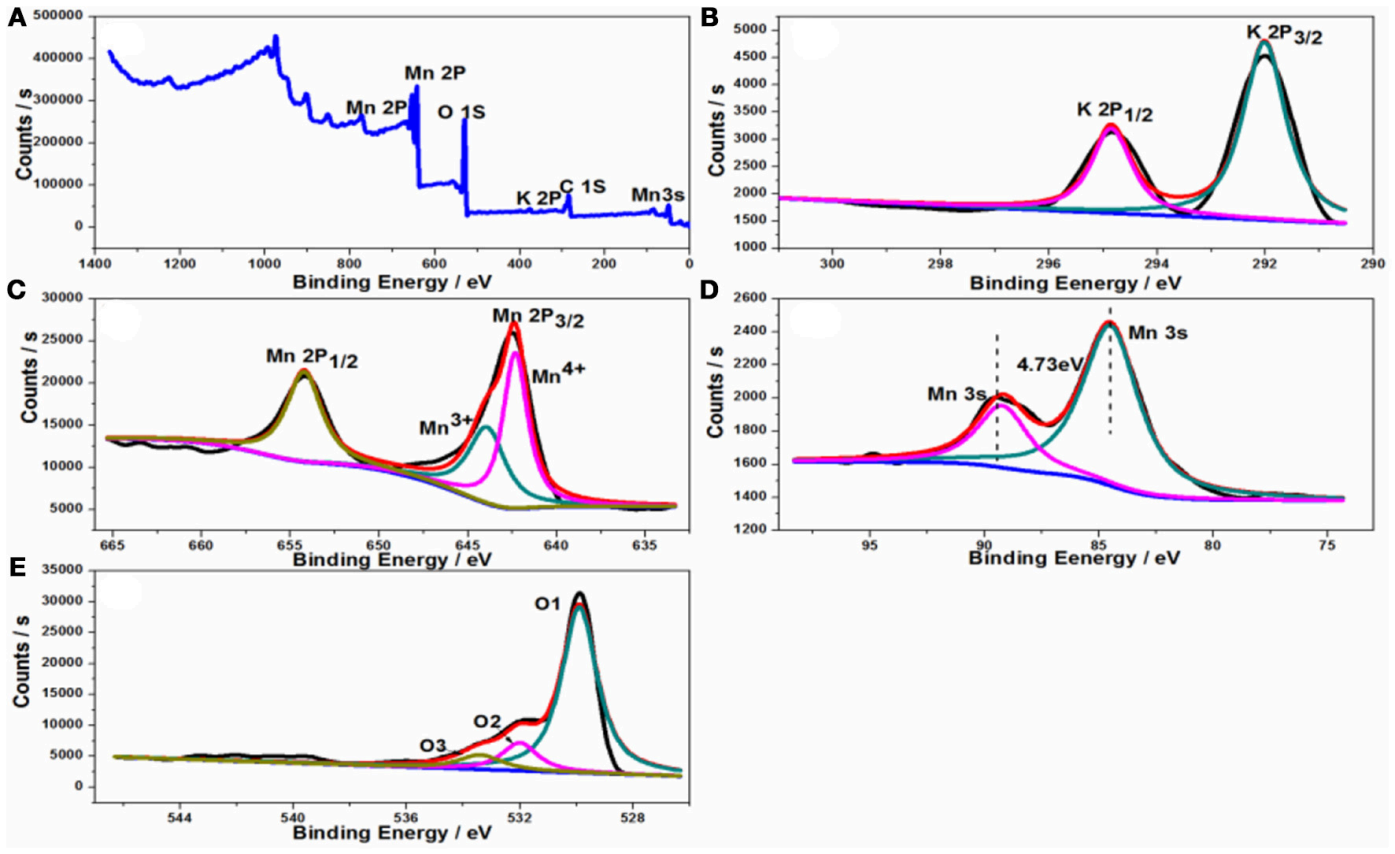
XPS spectra of the KMn_8_O_16_ sample: **(A)** survey spectrum; **(B)** K 2p spectrum; **(C)** Mn 2p spectrum; **(D)** Mn 3s; **(E)** O 1s spectrum.

The oxidation states and the composition of the samples were examined by XPS, and the spectra obtained were fitted with the XPS peak software. As shown in Figure 4A, the full scanning spectrum of tetragonal KMn_8_O_16_ shows that the presence of K, Mn, and O elements, which is in accordance with the EDS results. The K/Mn atomic ratios on the surface of sample is 0.135, which is very close to the bulk K/Mn atomic ratios measured by ICP, demonstrating that potassium is well dispersed in the sample. Thus, the chemical formula of the product is KMn_8_O_16_. In Figure 4B it can be noted that there are two separate peaks with the binding energies of 292.03 and 294.83 eV respectively, attributed to K2p_1/2_ and K2p_3/2_, and the binding energy difference between these two peaks of 2.8 eV, which is consistent with the previous reports. In addition, the high resolution spectra of Mn 2p in the as-prepared sample in Figure 4C show the binding energies (BEs) of the Mn 2p_1/2_ and Mn 2p_3/2_ peaks located at 654.18 and 642.48 eV, and the Mn2p_3/2_ binding energy of KMn_8_O_16_ are between that of the Mn_2_O_3_ and MnO_2_ powder standard, indicating that the existence of multiple Mn valences ions, which can be decomposed into two peaks of Mn^3+^ at 643.92 eV and Mn^4+^ at 642.33 eV. It is well-known that the separation of peak energies (ΔE) of Mn 3s can estimate the average oxidation state (AOS) of Mn, which were calculated from the ΔE of Mn 3s peaks {AOS = 8.956–1.126 × ΔE(3s)}. Thus, the calculated AOS of Mn is about 3.6 based on the Mn 3s splitting energy (4.73 eV) in Figure 4D. In addition, the high resolution O 1s peak is deconvoluted to three sub-peaks in Figure 4E, demonstrating three kinds of oxygen atoms in the sample, of which the fitting peak at 529.9 eV represents the typical Mn-O-Mn lattice oxygen, the fitting peak at 532.0 eV is assigned to Mn-OH surface hydroxyls or defect-oxide, while the peak at 533.4 eV is associated with the oxygen in the OH group adsorbed water.

**Figure 5:**
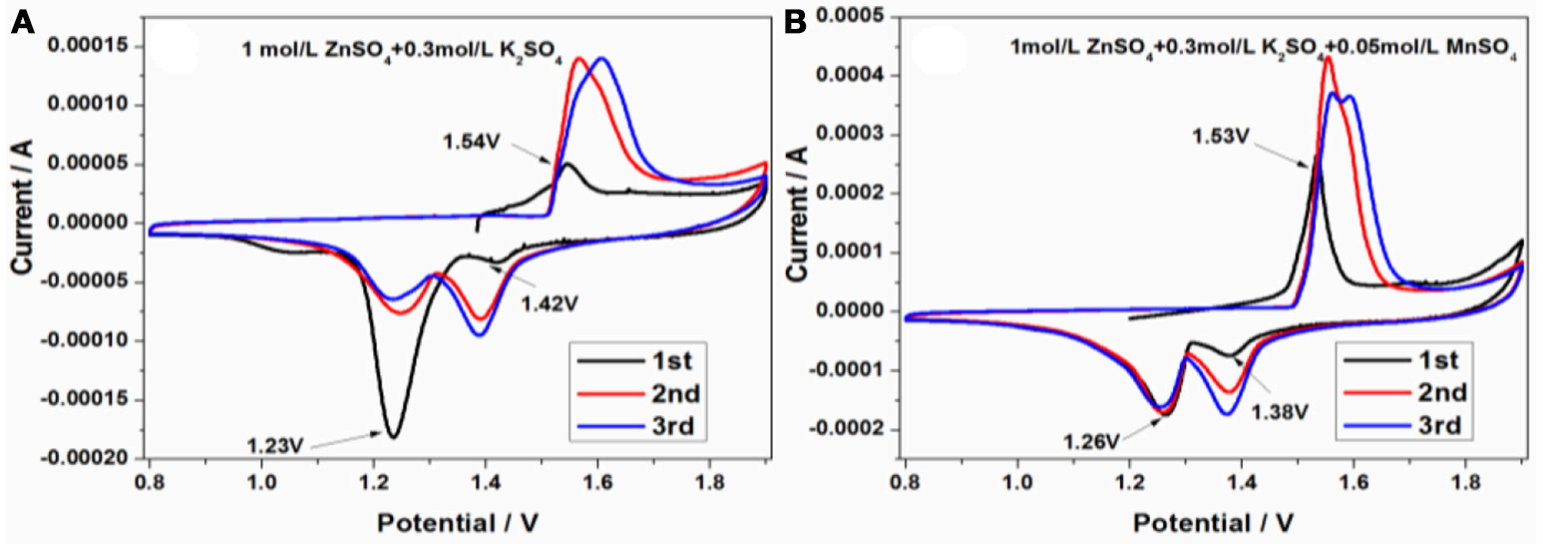
The cyclic voltammetry curves of Zn/KMn_8_O_16_ based on 1 mol/L ZnSO_4_ and 0.3 mol/L K_2_SO_4_ **(A)** without and **(B)** with 0.05 mol/L MnSO_4_ at a scan rate of 1 mV/s.

In order to evaluate the charge/discharge behavior in 1 mol/L ZnSO_4_ and 0.3 mol/L K_2_SO_4_ without or with 0.05 mol/L MnSO_4_, the cyclic voltammetry (CV) curves in the range of 0.8~1.9 V were conducted at a sweep rate of 1 mV/s. As is shown in Figure 5A, there is a small oxidation peak at 1.54 V and two reduction peaks, a very smaller reduction peak situated at 1.42 V and a larger reduction peak located at 1.23 V, respectively, indicating the strange initial charge/discharge process in 1 mol/L ZnSO_4_ and 0.3 mol/L K_2_SO_4_. That's to say, the initial coulombic efficiency exceeds 100%. However, apparently different from that shown in Figure 5A, after adding 0.05 mol/L MnSO_4_ into electrolyte in Figure 5B, the initial oxidation peak area is increased, the reduction peak area at 1.26 V tends to decrease, and the other of reduction peak at about 1.0 V disappear during the cathodic sweeping process, indicating the much better cyclic reversibility than that in Figure 5A. Notably, the position and CV profile in the subsequent sweeping curves are close to that in the first one. One cathodic peak at about 1.26 V nearly overlap, and the peak area of another cathodic peak at about 1.38 V seems to increase, demonstrating that the discharge capacity increases gradually with the cycle number increasing.

**Figure 6:**
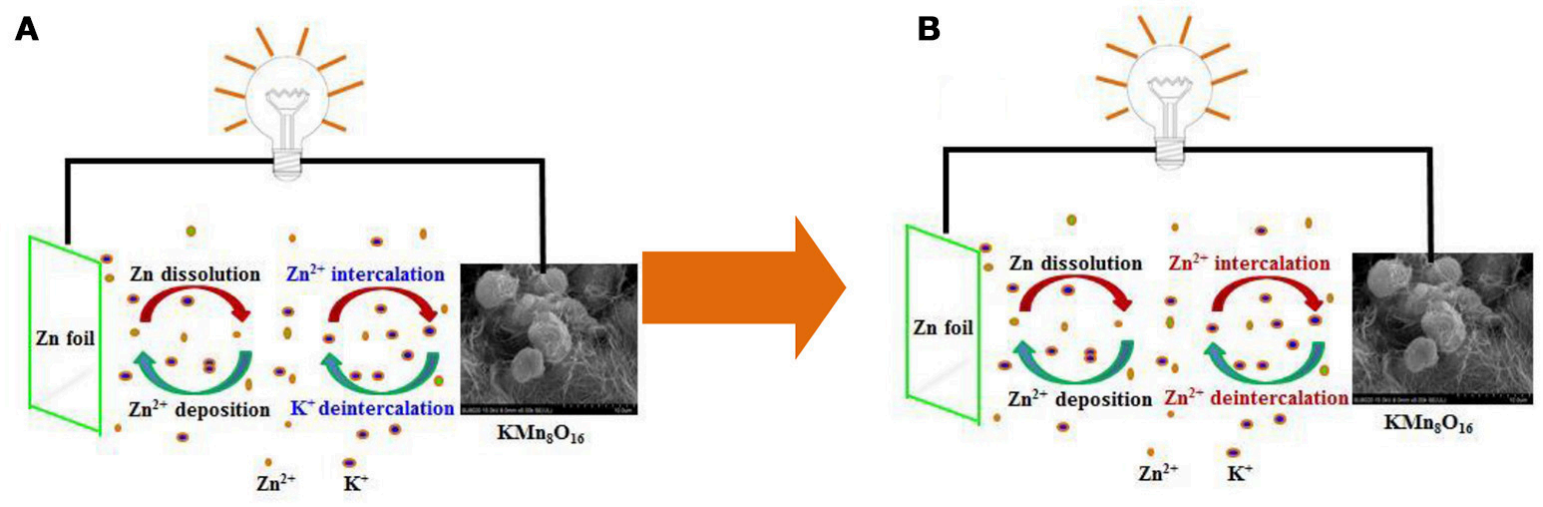
Schematic illustration of **(A)** the initial and **(B)** the subsequent charge/discharge process for Zn/KMn_8_O_16_.

In fact, the schematic illustration of charge/discharge mechanism for Zn/KMn_8_O_16_ hybrid aqueous battery in Figure 6 is a little different from those in previous reports by our groups (Wu et al., 2015, 2017). K^+^, Zn^2+^ and Mn^2+^ co-exist in the electrolyte. Based on the investigation on Na_3_V_2_(PO_4_)_3_ for aqueous zinc ion battery in Huang's group and the similarity of K^+^ and Na^+^ (Li et al., 2016), it can be inferred that potassium ions are de-intercalated from KMn_8_O_16_ and dissolved into the electrolyte quickly during the initial charge process. In the anode side, zinc ions in the electrolyte accept two electrons and deposit on the current collector. However, the reversible process is different from the previous process during the discharge process. It means that the intercalation of little potassium ions on the cathode will happen during the initial discharge process, accompanied mainly by the abundant intercalation of zinc ions. The main reason is that the radius of a zinc ion 0.6 Å is much smaller than that of potassium ions 1.33 Å, and the zinc ion can be intercalated easily into the MnO_2_-host structure. Thus, their electrode and total reactions on the positive side can be simply demonstrated as follows.

During the initial charge process, the equation of K^+^ deintercalation can be stated as follows.

(1) $$\begin{array}{r} KMn_{8}O_{16}\Leftrightarrow xK^{+}+K_{1-x}Mn_{8}O_{16}+xe^{-} \end{array}$$

While during the reversible intercalation of metal ion, the equation on the positive side can be described as follows.

(2) $$\begin{array}{r} 0.5xZn^{2+}+K_{1-x}Mn_{8}O_{16}+xe^{-}\Leftrightarrow Zn_{0.5x}K_{1-x}Mn_{8}O_{16} \end{array}$$

**Figure 7:**
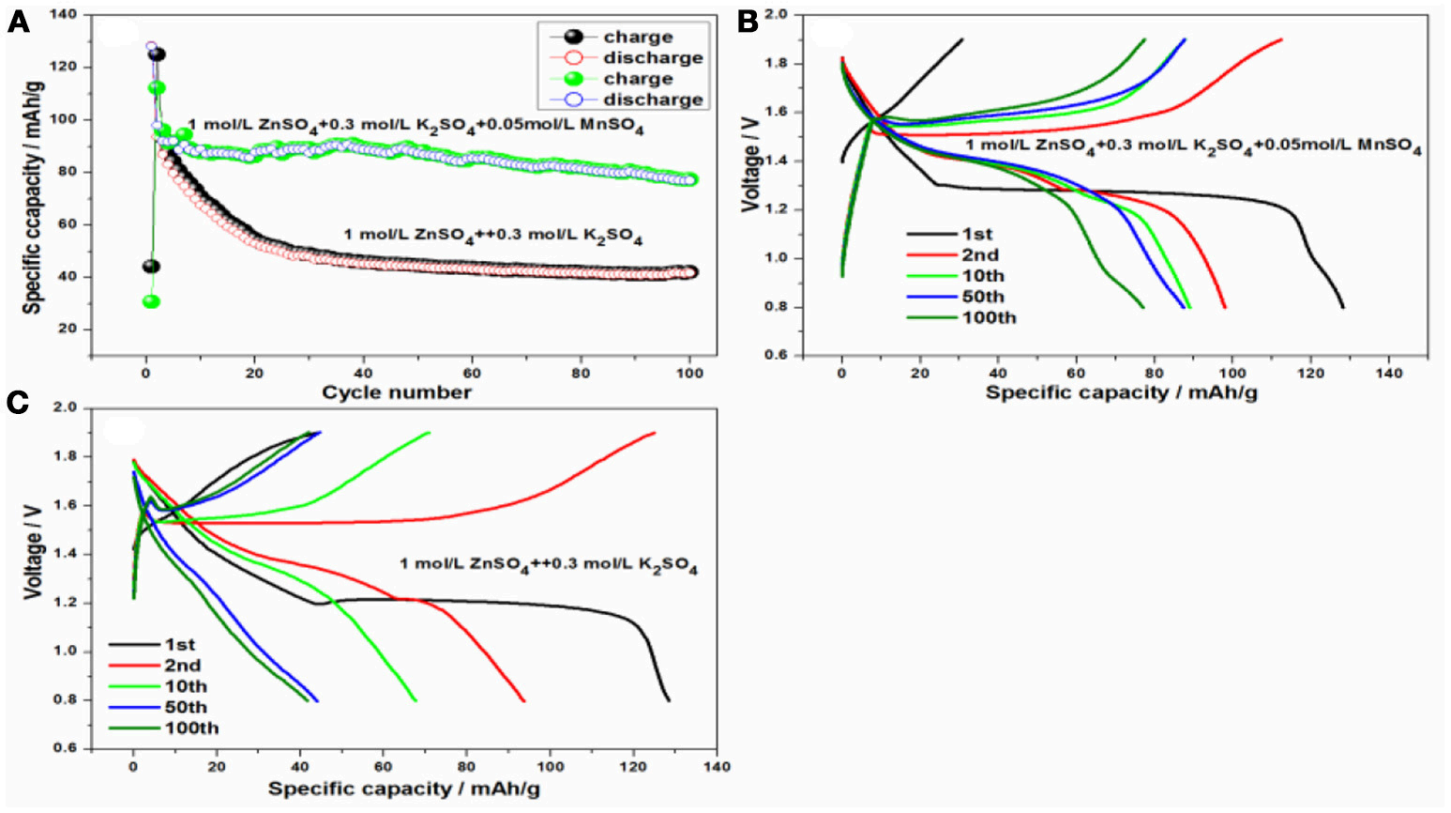
**(A)** The cycling performance of Zn/KMn_8_O_16_ and the charge/discharge curves based on 1 mol/L ZnSO_4_ and 0.3 mol/L K_2_SO_4_ at 100 mA/g **(B)** without and **(C)** with 0.05 mol/L MnSO_4_.

To better clarify the excellent cycling and rate stability of KMn_8_O_16_ material, the hybrid aqueous battery system was established based on KMn_8_O_16_ as the cathode and zinc as the anode, and the galvanostatic charge/discharge curves of the electrode were measured in Figure 7. Interestingly, all of the phenomenon can be confirmed from the cycling and charge/discharge curves at 1*C*-rate. The initial coulombic efficiency is as high as 290.5% whether adding 0.05 mol/L MnSO_4_ into 1 mol/L ZnSO_4_ and 0.3 mol/L or not in Figures 7B,C, which is consistent with the CV results. However, the cycling performance of battery with 0.05 mol/L MnSO_4_ is much better than that without MnSO_4_ in Figure 7A, and the discharge capacity of the former is still up to 77.0 mAh/g even after 100 cycles, which is much higher than that 41.7 mAh/g of the latter. The main reason is that the addition of MnSO_4_ has inhibited Mn dissolution, decreased the polarization overpotential and facilitated zinc dissolution (Wu et al., 2017), which has improved the cycleability of the electrode.

**Figure 8:**
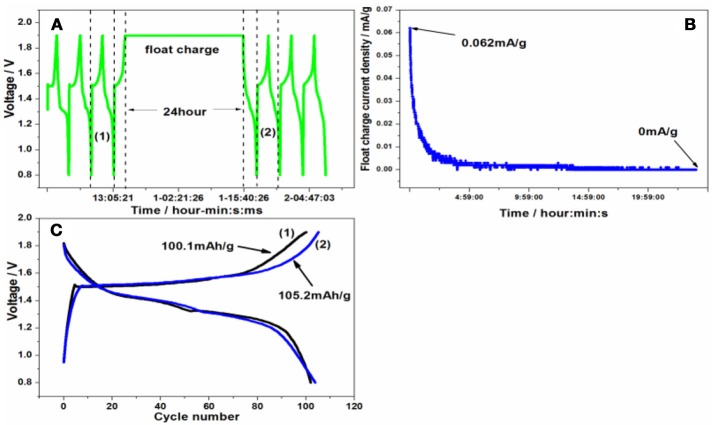
**(A)** The float charge procedure, **(B)** the float charge current density and **(C)** the charge/discharge curve of Zn/Zn/KMn_8_O_16_ battery before and after float charge.

**Figure 9:**
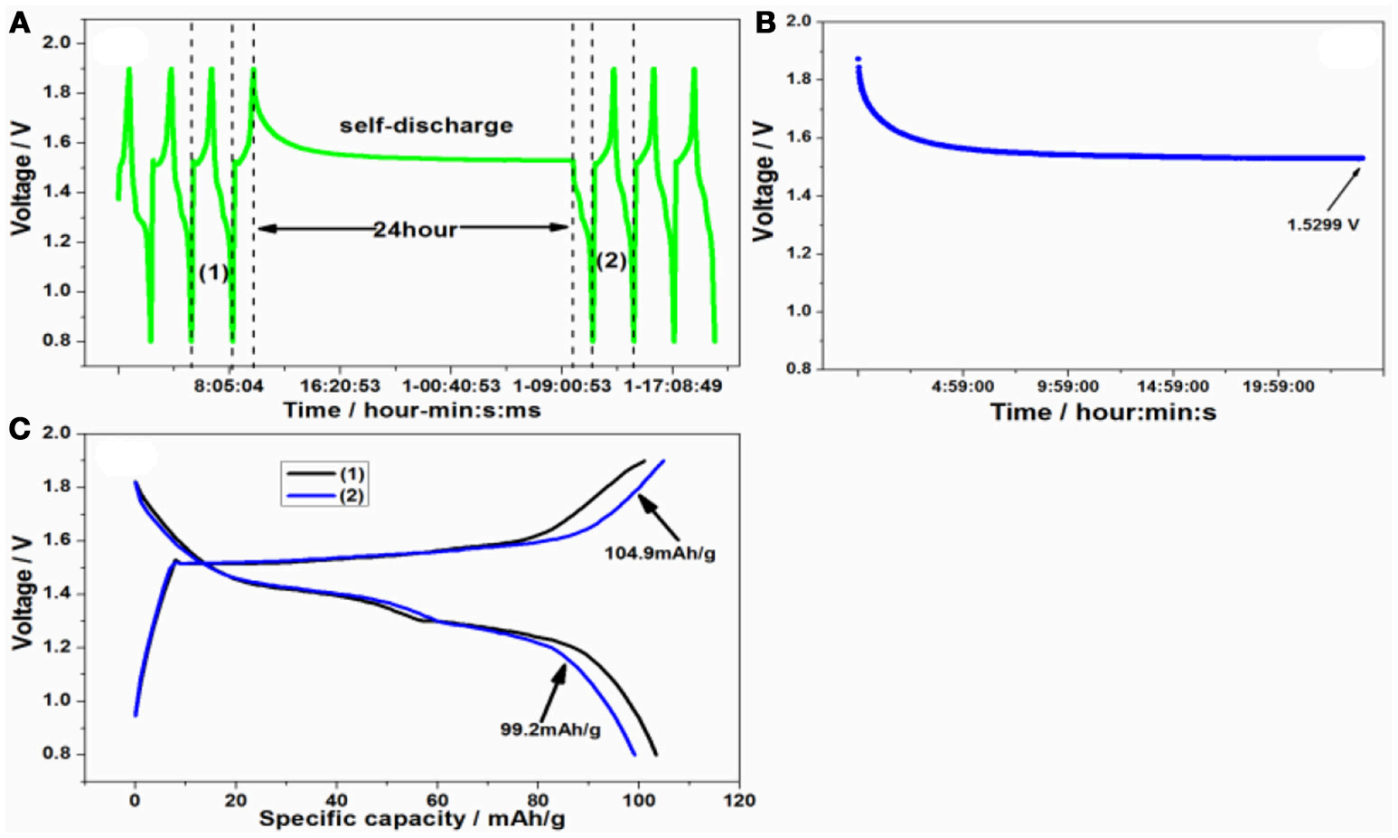
**(A)** The self-discharge procedure, **(B)** the voltage change after standing and **(C)** the charge/discharge curve of Zn/Zn/KMn_8_O_16_ battery before and after self-discharge.

A series of side reactions about electrodes usually happen (Wu et al., 2016). Here the float charge and self-discharge were evaluated. The battery was cycled for three times at 50 mA/g firstly, and then charged to 1.9 V, standing for 24 h or going on charging at constant voltage at room temperature in Figures 8A, 9A. Lastly it was cycled for three times. As can be seen from Figure 8B, the float charge current density decreased gradually with the time increasing, and quickly approaches to 0 mA/g. Even after float charge in Figure 8C, there is not capacity fading upon discharge process. On the contrary, the charge capacity increases to 105.2 mAh/g after float charge. During the self-discharge curve in Figure 9B, the voltage decreased to 1.5299 V. However, there is no much capacity fading in Figure 9C upon discharge process. That's to say, the discharge capacity can nearly return to the original level, indicating the smaller self-discharge phenomenon.

## Conclusions

In conclusion, KMn_8_O_16_ microspheres were successfully synthesized by a modified hydrothermal method, which were characterized by XRD, FE-TEM, EDS, TEM, and XPS. The material of cryptomelane-type structure was constituted by one-dimensional nano rods. When used as cathode material for aqueous battery, it shows an excellent cycling performance with a reversible capacity of up to 77.0 mAh/g even after 100 cycles and the small self-discharge phenomenon. CV test indicates that it can be used as the cathode material for aqueous zinc ion batteries in potential large scale energy storage field. Therefore, the results presented here will provide an alternative for aqueous batteries with high safety, low cost and high power density.

## Author contributions

All authors listed have made a substantial, direct and intellectual contribution to the work, and approved it for publication.

### Conflict of interest statement

The authors declare that the research was conducted in the absence of any commercial or financial relationships that could be construed as a potential conflict of interest.
